# Medial calcification in the arterial wall of smooth muscle cell‐specific *Smpd1* transgenic mice: A ceramide‐mediated vasculopathy

**DOI:** 10.1111/jcmm.14761

**Published:** 2019-11-19

**Authors:** Owais M. Bhat, Xinxu Yuan, Chad Cain, Fadi N. Salloum, Pin‐Lan Li

**Affiliations:** ^1^ Department of Pharmacology and Toxicology School of Medicine Virginia Commonwealth University Richmond Virginia; ^2^ Division of Cardiology Department of Internal Medicine VCU Pauley Heart Center Virginia Commonwealth University Richmond Virginia

**Keywords:** acid sphingomyelinase, arterial medial calcification, small extracellular vesicles, smooth muscle cells

## Abstract

Arterial medial calcification (AMC) is associated with crystallization of hydroxyapatite in the extracellular matrix and arterial smooth muscle cells (SMCs) leading to reduced arterial compliance. The study was performed to test whether lysosomal acid sphingomyelinase (murine gene code: *Smpd1*)‐derived ceramide contributes to the small extracellular vesicle (sEV) secretion from SMCs and consequently leads to AMC. In *Smpd1* ^trg^/SM^cre^ mice with SMC‐specific overexpression of *Smpd1* gene, a high dose of Vit D (500 000 IU/kg/d) resulted in increased aortic and coronary AMC, associated with augmented expression of RUNX2 and osteopontin in the coronary and aortic media compared with their littermates (*Smpd1* ^trg^/SM^wt^ and WT/WT mice), indicating phenotypic switch. However, amitriptyline, an acid sphingomyelinase (ASM) inhibitor, reduced calcification and reversed phenotypic switch. *Smpd1* ^trg^/SM^cre^ mice showed increased CD63, AnX2 and ALP levels in the arterial wall, accompanied by reduced co‐localization of lysosome marker (Lamp‐1) with multivesicular body (MVB) marker (VPS16), a parameter for lysosome‐MVB interaction. All these changes related to lysosome fusion and sEV release were substantially attenuated by amitriptyline. Increased arterial stiffness and elastin disorganization were found in *Smpd1* ^trg^ */*SM^cre^ mice as compared to their littermates. In cultured coronary arterial SMCs (CASMCs) from *Smpd1* ^trg^/SM^cre^ mice, increased P_i_ concentrations led to markedly increased calcium deposition, phenotypic change and sEV secretion compared with WT CASMCs, accompanied by reduced lysosome‐MVB interaction. However, amitriptyline prevented these changes in P_i_‐treated CASMCs. These data indicate that lysosomal ceramide plays a critical role in phenotype change and sEV release in SMCs, which may contribute to the arterial stiffness during the development of AMC.

## 1 INTRODUCTION

Vascular calcification is the build‐up or accumulation of apatite calcium salts in the media and/or intima of arteries that has been associated with ageing, chronic kidney disease, diabetes mellitus and atherosclerosis.^1^ It has been reported that the arterial calcification pathology mimics the bone formation process and that the earliest phase involves the osteogenic differentiation of vascular SMCs.^1^, ^2^, ^3^, ^4^ Various human^5^ and animal^5^, ^6^ studies have reported that osteogenic conversion of SMCs in the medial region appears prior to mineralization in arterial medial calcification (AMC), suggesting a critical role for smooth muscle cell (SMCs) phenotypic transition in this vascular pathologic change.^7^ It is known that both intimal and medial vascular calcification may be mediated by a common mechanism, namely the large increases in extracellular vesicles (EVs) in the vascular interstitial space, in particular, the small extracellular vesicles (sEVs) (with size of 40‐100 or to 140 nm). These sEVs are mainly produced and secreted from arterial SMCs.^8^ However, role of sphingolipids (SLs) such as ceramide in particular lysosomal ceramide in SMCs and associated pathogenic role in AMC is still poorly understood.

In response to various physiological stimuli or a mineral imbalance, vascular smooth muscle cells (VSMCs) secrete sEVs or exosomes, which act to nucleate calcium phosphate (Ca/P) crystals in the form of hydroxyapatite.^2^, ^4^, ^9^ More recently, sphingolipid‐mediated signalling took a central stage in understanding the regulation of matrix vesicles (MVs) or exosome release and vascular calcification. It has been reported that activation of sphingomyelin phosphodiesterase 3 (SMPD3, neutral sphingomyelinase) and cytoskeletal rearrangements in synthetic VSMCs led to multivesicular body (MVB) trafficking and elevated exosome secretion, which is a hallmark of vascular calcification or related vascular diseases.^10^ Sphingolipids belong to class of lipids located in the plasma membrane and at intracellular organelle membranes, which not only have structural roles but also carry signalling function. Ceramide (CER) and sphingosine‐1‐phosphate (S1P) are the major SLs that act as signalling molecules, control various cellular processes, such as cell growth, adhesion, migration, senescence, cell death and inflammatory response.^11^, ^12^, ^13^ Notably, several SL metabolites have been associated with the development of several pathologies, including diabetes, cancer, microbial infections, neurological syndromes and cardiovascular disease.^14^, ^15^, ^16^ Our laboratory previously reported that acid sphingomyelinase (ASM) plays an important role in glomerular injury^17^, ^18^ and inflammasome activation.^19^, ^20^

Acid sphingomyelinase (ASM), a lysosomal enzyme, is present in lysosomes and secretory lysosomes, and its fusion with plasma membrane results in the release of ceramide on the outer leaflet of the plasma membrane that serves to re‐organize and cluster receptors and signalling molecules.^21^, ^22^ This re‐organization of receptors and associated signalling molecules mediates various effects of ceramides at the cellular level.^23^ Bianco et al^24^ in 2009 revealed that acid sphingomyelinase (ASM) is a key enzyme involved in P2X_7_‐dependent microvesicle biogenesis at the surface of glial cells (microglia and astrocytes) via activation of P38 MAP kinase. This causes translocation of ASM from lysosomes to the plasma membrane outer leaflet, where it catalyses CER formation from sphingomyelin (SM).^24^ Further, they observed that SM to CER conversion perturbs membrane curvature and fluidity, favouring budding of multivesicles.^25^ In the human macrophage cell line, U937, it was found that activation of ASM in response to oxidized LDL‐containing immune complexes contributes to the release of IL‐1β in association with exosomes,^26^ and inhibiting ASM pharmacologically (desipramine) or genetically (ASM siRNA) reduced exosome and IL‐1β secretion. Recently, a study reported that activation of ASM by cigarette smoke (CS) in lung endothelial cells leads to ceramide production in these cells which then results in the release of EVs. However, treatment with imipramine, a functional inhibitor of ASM or ASM deletion, markedly decreased CS‐induced EV production.^27^ These findings were validated by ASM (*Smpd1* ^−/−^) knockout mice which showed reduced levels of EVs in plasma following CS exposure, whereas mice with overexpressed ASM in endothelial cells display increased levels of circulating EVs. Li et al, in human macrophages, found that CS may promote microvesicle shedding through an ASM‐dependent pathway via activation of p38 MAPK.^24^, ^28^ It was shown that the increase in lysosomal ceramides causes lysosomal dysfunction through the activation of cathepsins.^29^ On contrary to the well‐characterized function of surface acid sphingomyelinase and ceramides, the role of lysosomal acid sphingomyelinase and ceramide is poorly understood. PDMP, a ceramide analogue, is a promising target for preventing cardiovascular diseases such as atherosclerosis and cardiac hypertrophy^30^, ^31^ and for suppressing osteoclastogenesis by inhibiting glycosphingolipid synthesis.^31^, ^32^ Ode et al^33^ findings demonstrated that PDMP reduced osteoblastic proliferation and pre‐osteoblastic cell differentiation by translocation of mTORC1 from late endosome/lysosome (LE/Ly) to the endoplasmic reticulum (ER), suggesting inhibition of mTORC1 activity. This inhibitory action of ceramide analogue allows normal lysosomal functioning or lysosomal activation and their fusion with MVBs. We report here that SMC‐specific overexpression of ASM leads to extensive AMC, phenotype change and arterial stiffness upon receiving high dose of vitamin D injections. Administration of amitriptyline, a pharmacological inhibitor of ASM, to these animals ameliorates AMC.

## 2 MATERIAL AND METHODS

### 2.1 Reagents and antibodies

Cholecalciferol (vitamin D3) (C9756, Sigma‐Aldrich). Mouse monoclonal antibody against α‐SMA (ab7817, Abcam), Cre (Cat No. 6905, Novagen EMD Millipore) and ceramide (MID 15B4, Enzo ALX‐804‐196‐T050). Goat polyclonal antibody against mouse ASM (sc‐ 9817, USA). Secondary antibodies are Alexa‐488 or Alexa‐555‐labelled (Life technologies). Runx2 (ab23981, Abcam), OSP (ab63856, Abcam), SM22‐α (ab14106, Abcam), VPS16 (Cat. No.17776‐1‐AP, Protein biotech group), CD63 (ab216130, Abcam), Annexin‐II (AnX2, ab41803, USA) and alkaline phosphatase (ALP, sc‐28904, Santa Cruz, USA). Rat monoclonal antimouse Lamp‐1 (ab25245, Abcam), Von Kossa staining kit (ab150687, Abcam) and Alizarin Red S Solution (TMS‐008‐C, EMD Millipore) were used for detection AMC.

### 2.2 Primary culture of mouse CASMCs and Alizarin Red S staining

Mouse CASMCs were isolated as described previously.^34^ CASMCs were cultured in Dulbecco's modified Eagle's medium (DMEM, Gibco), supplemented with 10% FBS (Gibco) and 1% penicillin‐streptomycin (Gibco) in humidified 100% air and 5% CO_2_ mixture at 37°C. Cells were prepared in 6‐well plates overnight, and 70%‐80% confluent cells were treated with or without high phosphate (P_i_) (3 mmol/L)^35^ and then incubated for 2 weeks for in vitro calcification model.^36^ CASMCs were rinsed with PBS, fixed with 4% paraformaldehyde for 15 minutes and washed three times with diH_2_O. Then, cells were incubated with 1 mL of 1% Alizarin Red S for 5 minutes, washed with diH_2_O and visualized using a phase microscope. CASMCs were also treated with ASM inhibitor amitriptyline (20 μmol/L)^37^ and incubated for 24 hours.

### 2.3 Real‐time PCR studies

The mRNA levels of osteopontin and RUNX2 were determined in the wild‐type and *Smpd1* ^trg^/SM^cre^ CASMCs by RT‐PCR. The following mouse‐specific primers were used as follows: β‐actin, sense 5′‐TCGCTGCGCTGGTCGTC‐3′ and antisense 5′‐GGCCTCGTCACCCACATAGGA‐3′; OSP, sense 5′‐ATTTGCTTTTGCCTGTTTGG‐3′ and antisense 5′‐CTCCATCGTCATCATCATCG‐‐3′; RUNX2 sense 5′‐CCAGATGGGACTGTGGTTACC‐3′ and antisense 5′‐ACTTGGTGCAGAGTTCAGGG‐3′. β‐Actin was used as internal control to normalize the expression of mRNA. Fold changes were calculated as follows: 2^−ΔΔ^ threshold cycle.^38^

### 2.4 Isolation of sEVs

Small extracellular vesicles were isolated by differential ultracentrifugation from CASMC culture medium as described previously.^10^ As mentioned above, after 70%‐80% confluence, CASMCs were incubated with or without P_i_ (3 mmol/L)^35^ and also with ASM inhibitor amitriptyline (20 μmol/L)^37^ for 24 hours. Cell medium was collected and centrifuged at 300 *g* at 4°C for 10 minutes to remove detached cells. Supernatant was collected and filtered through 0.22‐μm filters to remove contaminating apoptotic bodies, microvesicles and cell debris. sEVs were obtained by ultracentrifugation of the supernatant at 100 000 *g* for 90 minutes at 4°C (Beckman 70.1 T1 ultracentrifuge rotator). The sEV pellet was washed with the PBS, ultracentrifuged at 100 000 *g* and resuspended in 50 μL of ice‐cold PBS. Crude sEV‐containing pellets are ready or stored at −80°C for further use. For nanoparticle analysis, samples were diluted into PBS and then analysed.

### 2.5 Nanoparticle tracking analysis (NTA)

Nanoparticle tracking analysis (NTA) was used to analyse the CASMC‐derived sEV using the light scattering mode of the NanoSight LM10 (Nano Sight Ltd.).^39^ Samples were diluted in PBS, and 5 frames (30 seconds each) were captured for each sample with background level 10, camera level 12 and shutter speed 30. Captured video was analysed using NTA software (version 3.2 Build 16), and an average size distribution graph was plotted using PRISM software (GraphPad).^10^

### 2.6 Vitamin D‐mediated AMC mouse model

SMC‐specific *Smpd1* transgenic mice (*Smpd1* ^trg^/SM^cre^ sphingomyelin phosphodiesterase 1 [*Smpd1*]) were used in the present study. 12‐ to 14‐week‐old male C57BL/6J wild‐type, *Smpd1* ^trg^/SM^wt^ and *Smpd1* ^trg^/SM^cre^ mice were used. Characterization of mice was performed by genotyping, in vivo/ex vivo imaging and confocal microscopy. All protocols were approved by the Institutional Animal Care and Use Committee of Virginia Commonwealth University. Animals were further randomized into six groups for each mouse strain (WT/WT, *Smpd1* ^trg^/SM^wt^ and *Smpd1* ^trg^/SM^cre^) to receive the active vitamin D (Vit D) (500 000 IU/kg/bw/d) or matched vehicle (5% v/v ethanol) by subcutaneous injection for 3‐4 days. After 16‐17 days of post‐injection period, animals were sedated with 2% isoflurane that was provided through a nose cone. Blood samples were collected, and plasma was isolated and stored at −80°C. Mice were killed, and the heart and aorta were collected, with a portion stored in 10% buffered formalin for histopathological analysis and immunostaining. Another part of heart and aorta were frozen in liquid nitrogen and stored at −80°C for dual‐fluorescence staining and confocal analysis by making frozen tissue slides. Vit D‐induced mouse model is commonly used to investigate AMC.^40^ Normal C57BL/6N, *Smpd1* ^trg^/SM^wt^ or *Smpd1* ^trg^/SM^cre^ mice were injected with a high dose of Vit D (500 000 IU/Kg/bw/d) for 3‐4 days, and ASM inhibitor amitriptyline (10 mg/kg BW)^41^ was injected intraperitoneally (i.p) alternatively for 12 days (n = 5‐6 per group). The Vit D solution was prepared as follows: vitamin D3 (66 mg) dissolved in 200 μL of absolute ethanol was mixed with 1.4 mL of cremophor (Sigma‐Aldrich) at RT for 15 minutes, and then, 750 mg of dextrose dissolved in 18.4 mL of sterilized water was added at RT for 15 minutes. The Vit D solution was stored at 4°C till use, but usually made fresh after couple of days.

### 2.7 Alizarin Red S staining, Von Kossa staining and immunohistochemical analyses

Alizarin Red S staining was used to detect AMC. Paraffin‐embedded artery samples were deparaffinised with alcohol and xylene. After three washes with distilled water, the arteries were stained with 1% Alizarin Red S for 5 minutes and washed with acetone for 20 seconds followed by acetone‐xylene wash for further 20 seconds. The positively stained area showed a reddish colour. Von Kossa staining was performed to detect mineralization.^42^ Briefly, deparaffinized sections were washed in Milli‐Q water for 5 minutes, incubated in 1% aqueous silver nitrate solution under a UV lamp for 1 hour or more, and then washed with Milli‐Q water three times at RT. Non‐specific staining was removed by treating sections with 2.5% sodium thiosulphate for 5 minutes at RT. Finally, staining with nuclear stain haematoxylin, dehydrated and mounted with DPX. For histological analysis, paraffin sections (5 μm) were made with fixed mouse aortas and hearts. Immunohistochemical analyses were carried out as described previously^43^ or following the manufacturer's instructions for CHEMICON IHC Select HRP/DAB Kit (EMD Millipore). Briefly, after antigen retrieval, endogenous peroxidase activity was quenched using 3% H_2_O_2_. After blocking with 2.5% horse serum for 1 hour at RT, slides were incubated with indicated primary antibodies against SM22‐α (1:1500), RUNX2 (1:300), OSP (1:100), CD63 (1:50), AnX2 (1:50) and ALP (1:50) overnight at 4°C and then incubated with biotinylated secondary antibodies and a streptavidin peroxidase complex (PK‐7800, Vector Laboratories). The slides were sequentially treated according to the protocol described by the manufacturer. Finally, slides were counterstained with haematoxylin, dehydrated and mounted with DPX. The area percentage of the positive staining was calculated using Image‐Pro Plus 6.0 software.^44^

### 2.8 Immunofluorescence staining

Indirect immunofluorescent staining was used to determine co‐localization of the MVB marker (VPS16) vs lysosomal marker (Lamp‐1), which depicts MVB‐lysosome interaction in SMCs. Frozen aortic tissue slides were fixed in acetone and then incubated overnight with VPS16 (1:300) and Lamp‐1 (1:200) at 4°C. Double immunofluorescent staining was achieved by incubating with either Alexa‐488 or Alexa‐555‐labelled secondary antibodies for 1 hour at RT. After washing, slides were mounted with a DAPI‐containing mounting solution and then observed using confocal laser scanning microscope (FluoView FV1000, Olympus). As previously described,^44^ images were analysed by the Image‐Pro Plus 6.0 software (Media Cybernetics, Bethesda, MD), where co‐localization was measured and expressed as the Pearson correlation coefficient (PCC).^44^

### 2.9 Calcium and phosphate assay

Blood concentrations of calcium and phosphate were measured using commercially available kits ab102505, Abcam, USA, and ab65622, Abcam, USA, as described by manufacture's protocol.

### 2.10 Elastin staining

Elastin staining of the aortic tissues was performed using commercially available kits ab150667, Abcam, USA, as described by manufacture's protocol.

### 2.11 Non‐invasive, in vivo measurement of aortic pulse wave velocity (PWV)

Pulse wave velocity was measured 3 weeks post‐Vit D injection or vehicle using a high‐resolution Doppler ultrasound instrument (Vevo2100, Visual Sonics), as described.^45^ Briefly, 2% isoflurane was used to anaesthetize the mice prior to mounting on a heated (37°C) platform to monitor electrocardiogram (ECG), heart rate (HR), respiratory rate, and to eliminate movement artefacts. Abdominal hair was removed using hair removal cream (Veet). Isoflurane was reduced to 1.5% with slight adjustments in order to maintain a heart rate (HR) of approximately 450 bpm. Aorta was identified using a B‐mode cross‐sectional image, and flow wave Doppler measurements were taken longitudinally at two locations along the aorta, one proximal and one distal to the heart, at 30 MHz with a pulse‐repetition frequency of 40 kHz at a depth of 6 mm. There was no difference in the HR between the proximal and distal point measurements. In offline analysis of the images, the time (ms) from the peak of the ECG R wave to the foot of the flow waves at both the proximal and distal locations was measured, in at least 5 replicates for each location per mouse. The difference between proximal point and distal point arrival time yields the transit time (TT). Pulse wave velocity (mm/ms) was calculated from TT(Δt) and the distance between the measurement sites (Δd).

#### 2.11.1 Statistical analysis

All of the values are expressed as mean ± SEM. Significant differences among multiple groups were examined using two‐way ANOVA followed by Duncan's test. *P* < .05 was considered statistically significant.

## 3 RESULTS

### 3.1 Characterization of *Smpd1* ^trg^/SM^cre^ transgenic mice

**Figure 1:**
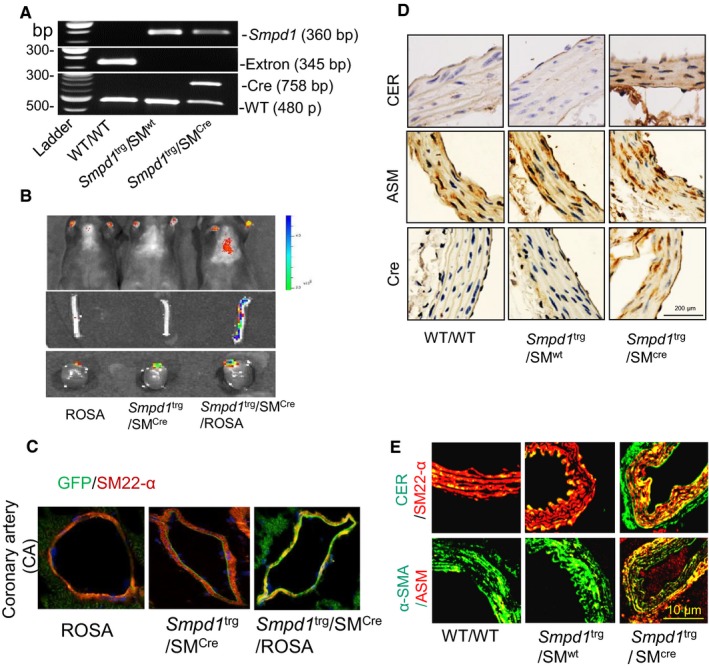
Characterization of SMC‐specific *Smpd1* transgenic mice. A, *Smpd1* ^trg^/SM^cre^ had 2 positive PCR products including 758 bp for Cre and 360 bp for transgenic *Smpd1* gene. *Smpd1* ^trg^/SM^wt^ mice had positive transgenic *Smpd1* gene (360 bp), but no Cre (758 bp). WT/WT (*Smpd1* ^trg^/SM^wt^) mice only had wild‐type *Smpd1* gene with transgenic *Smpd1* gene and Cre gene. B, Cre‐mediated SMC‐specific recombination was validated by breeding the *Smpd1* ^trg^/SM^cre^ mice with ROSA reporter mice; in vivo and ex vivo imaging of the offspring showed GFP expression in the aorta and heart. C, Representative fluorescent confocal microscopic images showed increased co‐localization of GFP (green) vs SM22‐α (red) in coronary arterial wall of *Smpd1* ^trg^/SM^cre^ mice. D, Representative immunohistochemical images of aortic sections revealed increased immunostaining of CER, ASM and Cre (brown stain) in *Smpd1* ^trg^/SM^cre^ as compared to their littermates. E, Representative fluorescent confocal microscopic images showed increased co‐localization of α‐SMA (green) vs ASM (red, acid sphingomyelinase) and SM22‐α (red) vs ceramide (green) in *Smpd1* ^trg^/SM^cre^ mice compared with their littermates (*Smpd1* ^trg^/SM^wt^ and WT/WT mice). ASM, acid sphingomyelinase; CER, Ceramide; Cre, Cre recombinase protein; SM22‐α, SMC lineage marker; SMC, smooth muscle cell; α‐SMA, α‐smooth muscle cell actin (smooth muscle cell marker)

To confirm the role of sphingolipid, ceramide in AMC, we employed an animal model, namely SMC‐specific *Smpd1* transgenic mice (*Smpd1* ^trg^/SM^cre^) which caused overexpression of *Smpd1* gene in SMCs that encodes acid sphingomyelinase (ASM) to produce ceramide via hydrolysis of sphingomyelin. *Smpd1* ^trg^/SM^cre^ were generated by crossing the *Smpd1* gene knock‐in mice (with floxed blocker for its transcription)^46^ and SM22α‐Cre transgenic mice (from Jackson laboratory, B6.129S6‐ *Tagln ^tm2(cre)Yec^*/J, Stock No: 006878; SM22α‐creKI). Figure 1A presents the genotyping gel documents, which show 3 PCR products including 360 bp for *Smpd1* transgene allele, 345 bp for HRPT exon as marker of WT allele and 758 bp for Cre. To confirm the specific overexpression of *Smpd1* gene in the SMCs, each batch of *Smpd1* ^trg^/SM^cre^ mice was crossed with ROSA mice with floxed GFP (from Jackson laboratory, B6.129S6‐ *Tagln^tm2(cre)Yec^*/J, Stock No: 006878; SM22α‐creKI) to produce *Smpd1* ^trg^/SM^cre^/ROSA mice, which showed green fluorescence in the SMCs indicating SMC‐specific overexpression of *Smpd1* gene (Figure 1B). We observed co‐localization of GFP with SM22‐α in the coronary arterial wall of *Smpd1* ^trg^/SM^cre^/ROSA mice (Figure 1C). We observed markedly increased immunostaining of CER, ASM and Cre in the aortic wall of *Smpd1* ^trg^/SM^cre^ mice as compared to *Smpd1* ^trg^/SM^wt^ and WT/WT mice (Figure 1D). As shown in Figure 1E**,** in SMC‐specific *Smpd1* transgenic mice, largely increased co‐localization (yellow spots) of α‐SMA (green) vs ASM (red) or ceramide (green) vs SM22‐α (red) was observed in *Smpd1* ^trg^/SM^cre^ mice compared with *Smpd1* ^trg^/SM^wt^ and WT/WT mice.

### 3.2 Aortic calcification and SMC phenotype transition in Vit D‐treated *Smpd1* ^trg^/SM^cre^ mice

**Figure 2:**
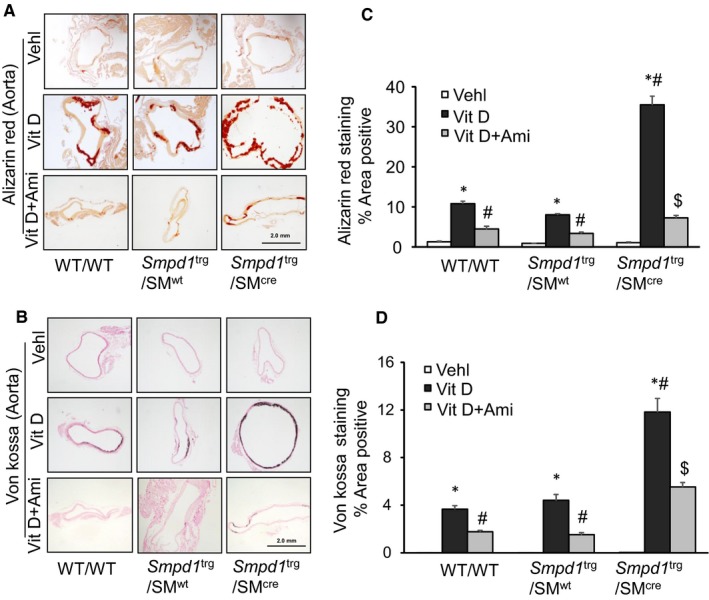
Aortic calcification in Vit D‐treated SMC‐specific *Smpd1* transgenic mice. Representative images of aortic sections stained by A, Alizarin Red S (red colour) and B, Von Kossa (black colour) staining showed significantly increased calcification in the aortic media of Vit D‐treated *Smpd1* ^trg^/SM^cre^ mice as compared to their littermates which was reduced by amitriptyline. C, D, Summarized data showing calcification in the aortic media. Data are shown as mean ± SEM of values n = 6. Ami, amitriptyline; SMC, smooth muscle cell; Vehl, vehicle; Vit D, vitamin D. *P ˂ .05 vs WT/WT Vehl; #*P* ˂ .05 vs WT/WT Vit D group; $*P* ˂ .05 vs* Smpd1* ^trg^/SM^cre^ Vit D group by two‐way ANOVA followed by Duncan's test

Both Alizarin Red S and Von Kossa staining showed more significantly enhanced calcification in the aortic medial wall of Vit D‐treated *Smpd1* ^trg^/SM^cre^ mice as compared to their littermates (*Smpd1* ^trg^/SM^wt^ and WT/WT mice) which was significantly decreased by amitriptyline (Figure 2A,B). As summarized in the bar graphs (Figure 2C,D), overexpression of *Smpd1* gene in arterial smooth muscle led to severe calcification upon s.c injection of high dose of Vit D (500 000 IU/kg/d) while as inhibition of ASM with amitriptyline, a pharmacological inhibitor of ASM, reduced arterial calcification. Also, we observed significantly decreased arterial calcification in Vit D‐treated *Smpd1* KO mice as compared to WT/WT mice as shown in Figure S1A and B, indicating that both genetic and pharmacological inhibition of ASM reduced arterial calcification.

**Figure 3:**
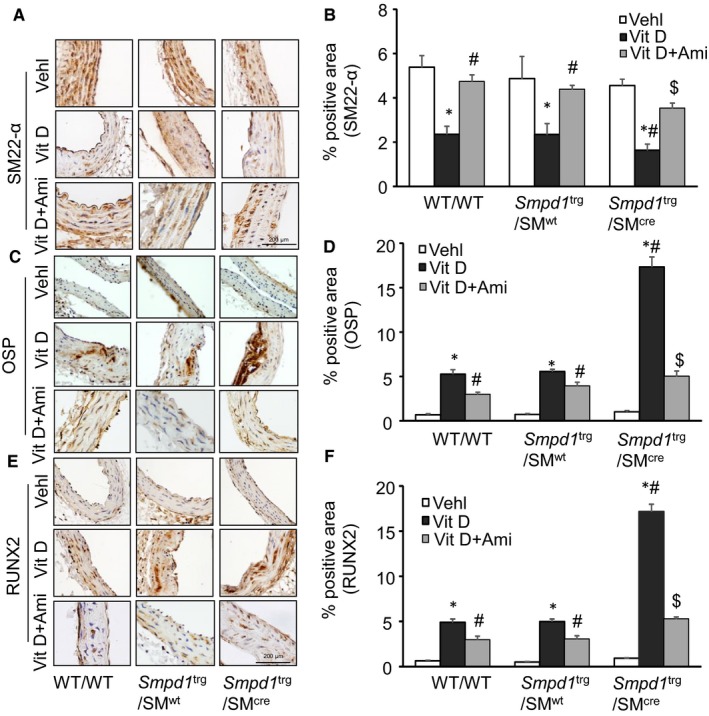
Aortic SMC phenotype changes in Vit D‐treated SMC‐specific *Smpd1* transgenic mice. Representative immunohistochemical images from the aorta show AMC associated with A, B, decreased expression of SM22‐α (brown stain); C, D, increased expression of OSP; and E, F, RUNX2 (brown stain) in the aortic media of *Smpd1* ^trg^/SM^cre^ mice receiving Vit D injections as compared to their littermates which was prevented by amitriptyline treatment. Data are shown as mean ± SEM of values. n = 6. Ami, amitriptyline; OSP, osteopontin; RUNX2, runt‐related transcription factor 2; SMC, smooth muscle cell; Vehl, vehicle; Vit D, vitamin D. *P ˂ .05 vs WT/WT Vehl; #*P* ˂ .05 vs WT/WT Vit D group; $*P* ˂ .05 vs* Smpd1* ^trg^/SM^cre^ Vit D group by two‐way ANOVA followed by Duncan's test

The present study also showed that phenotypic switch in SMCs during AMC was characterized by decreased expression of the VSMC lineage marker, smooth muscle 22α (SM22‐α) (Figure 3A) and up‐regulation of both OSP (Figure 3C) and RUNX2 (Figure 3E) while as amitriptyline treatment prevented this phenotype change. As shown in the bar graph (Figure 3B), it is clear that SM22‐α expression significantly decreased in the aortic medial wall of Vit D‐treated *Smpd1* ^trg^/SM^cre^ mice as compared to their littermates (*Smpd1* ^trg^/SM^wt^ and WT/WT mice). However, the expression of OSP and RUNX2 significantly increased in these transgenic mice as compared to their littermates (Figure 3D,F). However, inhibition of ASM by amitriptyline which blocked the formation of ceramide from sphingomyelin prevented the phenotype change in arterial SMCs during AMC. Together, these results suggested that sphingolipid synthesis may be associated with the development of vascular calcification and phenotype change.

### 3.3 Coronary arterial calcification and SMC phenotype change in Vit D‐treated *Smpd1* ^trg^/SM^cre^ mice

**Figure 4:**
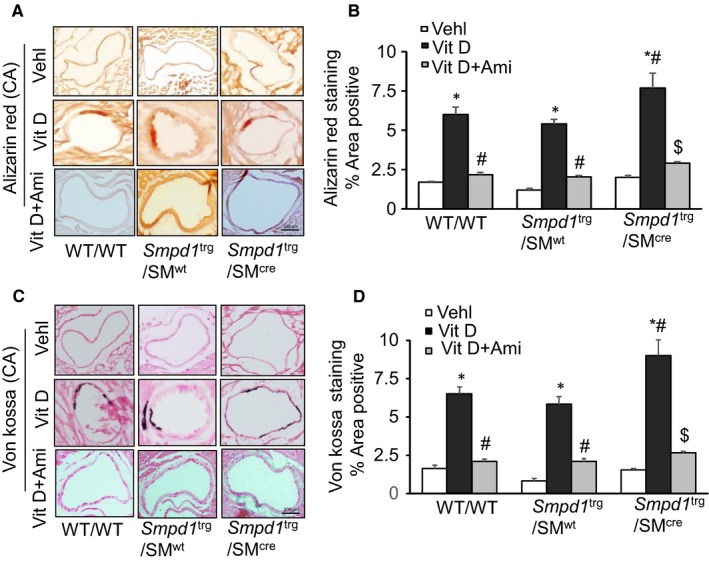
Coronary arterial calcification in Vit D‐treated SMC‐specific *Smpd1* transgenic mice. Representative images of coronary artery sections showed coronary AMC by A. Alizarin Red S (red colour) and C. Von Kossa (black colour) staining in Vit D‐treated mouse model. B, D, The bar graphs show a significantly increased calcification staining in *Smpd1* ^trg^/SM^cre^ mice as compared to their littermates (*Smpd1* ^trg^/SM^wt^ and WT/WT mice) which was significantly reduced by amitriptyline. Data are shown as mean ± SEM of values. n = 6. Ami, amitriptyline; CA, coronary artery; SMC, smooth muscle cell; Vehl, vehicle; Vit D, vitamin D. *P ˂ .05 vs WT/WT Vehl; #*P* ˂ .05 vs WT/WT Vit D group; $*P* ˂ .05 vs* Smpd1* ^trg^/SM^cre^ Vit D group by two‐way ANOVA followed by Duncan's test

**Figure 5:**
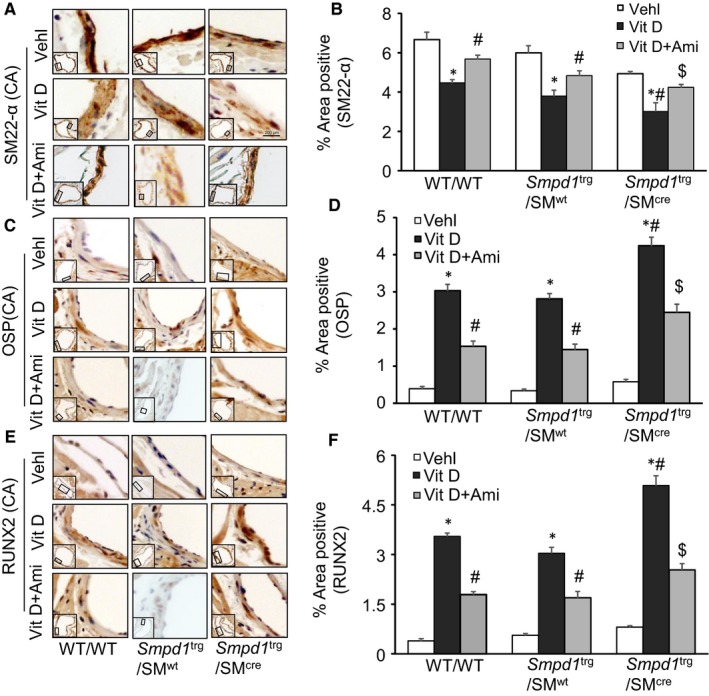
Coronary arterial smooth muscle phenotype changes in Vit D‐treated SMC‐specific *Smpd1* transgenic mice. Representative images of coronary artery sections showed decreased expression of A. SM22‐α (brown stain) and increased expression of C. OSP; and E. RUNX2 (brown stain) in the coronary arterial wall of Vit D‐treated *Smpd1* ^trg^/SM^cre^ mice as compared to their littermates which was switched by amitriptyline. B, D, F, The data of SMC phenotypic transition are summarized in the bar graph. Data are shown as mean ± SEM of values. n = 6. Ami, amitriptyline; OSP, osteopontin; RUNX2, runt‐related transcription factor 2; SM22‐α, smooth muscle cell marker; SMC, smooth muscle cell; Vehl, vehicle; Vit D, vitamin D. *P ˂ .05 vs WT/WT Vehl; #*P* ˂ .05 vs WT/WT Vit D group; $*P* ˂ .05 vs* Smpd1* ^trg^/SM^cre^ Vit D group by two‐way ANOVA followed by Duncan's test

In addition to the aortic medial calcification, we extended our approach to find AMC and SMC phenotypic changes in coronary arteries. We observed an increased AMC by Alizarin Red S and Von Kossa staining in the coronary arterial wall of Vit D‐treated *Smpd1* ^trg^/SM^cre^ mice as compared to their littermates (*Smpd1* ^trg^/SM^wt^ and WT/WT mice) which was reduced by amitriptyline (Figure 4A,C). The bar graph shows a significant increase in Vit D‐induced calcification in *Smpd1* ^trg^/SM^cre^ mice as compared to their littermates which was significantly decreased by amitriptyline (Figure 4B,D). Moreover, amitriptyline prevented the Vit D‐induced decreased expression of SM22‐α (Figure 5A) and increased OSP and RUNX2 (Figure 5C,E) in the coronary arterial wall of *Smpd1* ^trg^/SM^cre^ mice as compared to their littermates. As shown in Figure 5B,D,F, the SMC phenotype was changed in coronary arterial media towards more dedifferentiated or osteogenic status when *Smpd1* gene was specifically overexpressed in these SMCs, which was prevented due to ASM inhibition by amitriptyline.

### 3.4 Lysosome‐MVB interactions and sEV release in the arterial wall of Vit D‐treated SMC‐specific *Smpd1* transgenic mice

**Figure 6:**
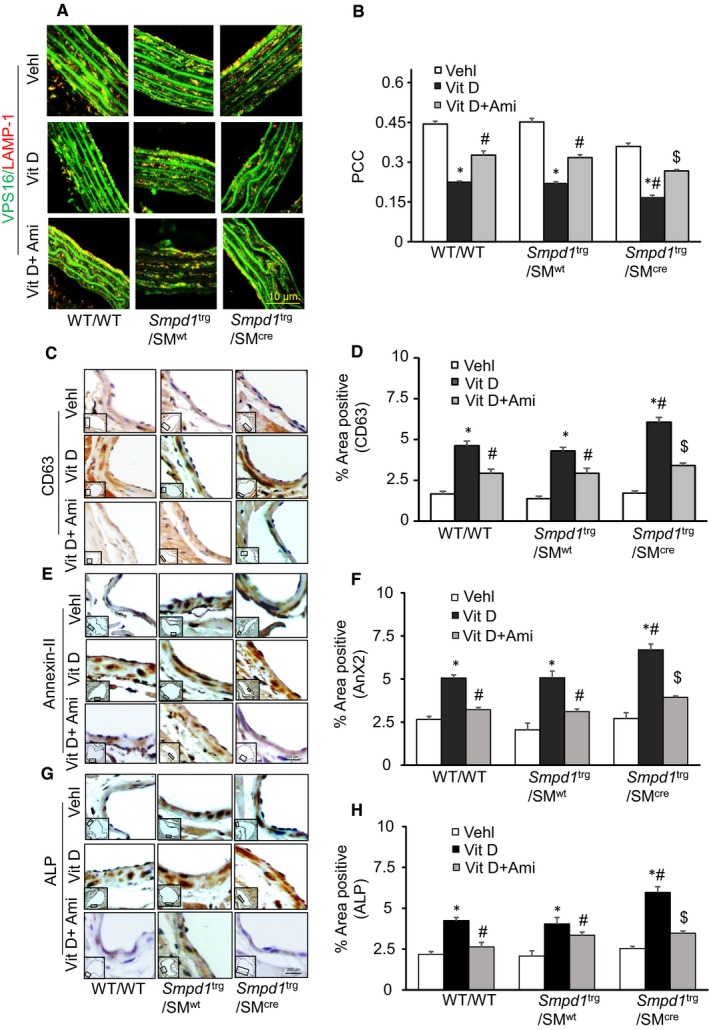
Lysosome‐MVB changes and sEV secretion in Vit D‐treated SMC‐specific *Smpd1* transgenic mice. A, Representative confocal microscopic images showed that co‐localization of MVBs (VPS16, green) and lysosomes (lysosome marker, Lamp‐1, red) in SMCs was much lower in the aortic media of *Smpd1* ^trg^/SM^cre^ mice than their littermates (*Smpd1* ^trg^/SM^wt^ and WT/WT mice) receiving Vit D injection while as amitriptyline significantly increased the co‐localization coefficient of these markers. B, The co‐localization coefficient (PCC) as shown in the bar graph. Representative immunohistochemical images showed that sEV makers C. CD63 (brown stain), E. AnX2 and G. ALP staining were significantly increased in the coronary arterial media of Vit D‐treated *Smpd1* ^trg^/SM^cre^ mice than their littermates which was decreased by amitriptyline. Summarized data showing area percentage of D. CD63, F. AnX2 and H. ALP‐positive staining in coronary arterial wall. Data are shown as mean ± SEM of values. n = 6. ALP, alkaline phosphatase; AnX2, Annexin‐II; PCC, Pearson correlation coefficient. *P ˂ .05 vs WT/WT Vehl; #*P* ˂ .05 vs WT/WT Vit D group; $*P* ˂ .05 vs* Smpd1* ^trg^/SM^cre^ Vit D group by two‐way ANOVA followed by Duncan's test

The literature reports that sphingolipids such as CER participate in exosome or sEV biogenesis, formation of MVBs and their fusion with plasma membrane causing increased exosome secretion.^47^ We observed that co‐localization of MVBs (VPS16, green) and lysosomes (Lamp‐1, red) in SMCs was much lower in the aortic medial wall of SMC‐specific *Smpd1* transgenic mice than their littermates (*Smpd1* ^trg^/SM^wt^ and WT/WT mice) receiving Vit D injection, while as amitriptyline enhanced this interaction between MVBs (VPS16, green) and lysosomes (Lamp‐1, red) in SMCs (Figure 6A). The co‐localization coefficient (PCC) of both markers in *Smpd1* ^trg^/SM^cre^ mice was clearly reduced as shown in the bar graph, while as amitriptyline significantly increased the co‐localization coefficient of these markers (Figure 6B). Immunohistochemically, we indeed found that CD63 (Figure 6C), Annexin‐II (AnX2) (Figure 6E) and alkaline phosphatase (ALP) (Figure 6G) staining as sEV markers were significantly increased in the coronary arterial wall of Vit D‐treated *Smpd1* ^trg^/SM^cre^ mice than their littermates (*Smpd1* ^trg^/SM^wt^ and WT/WT) which were decreased by amitriptyline as shown in the bar graphs (Figure 6D,F,H). This suggests that more MVBs may not able to fuse with lysosomes, increasing sEV secretions during AMC. Together, these results indicated that increased ceramide levels in lysosomes due to SMC‐specific overexpression of *Smpd1* may result in reduced lysosome‐MVB interactions, and enhanced release of sEVs. However, preventing the increased production of lysosomal ceramide via inhibition of ASM by amitriptyline in *Smpd1* ^trg^/SM^cre^ mice may result in the reduced sEV secretion during AMC.

### 3.5 Enhanced calcification and phenotype change in the P_i_ *‐*treated *Smpd1* ^trg^/SM^cre^ mice CASMCs in vitro

**Figure 7:**
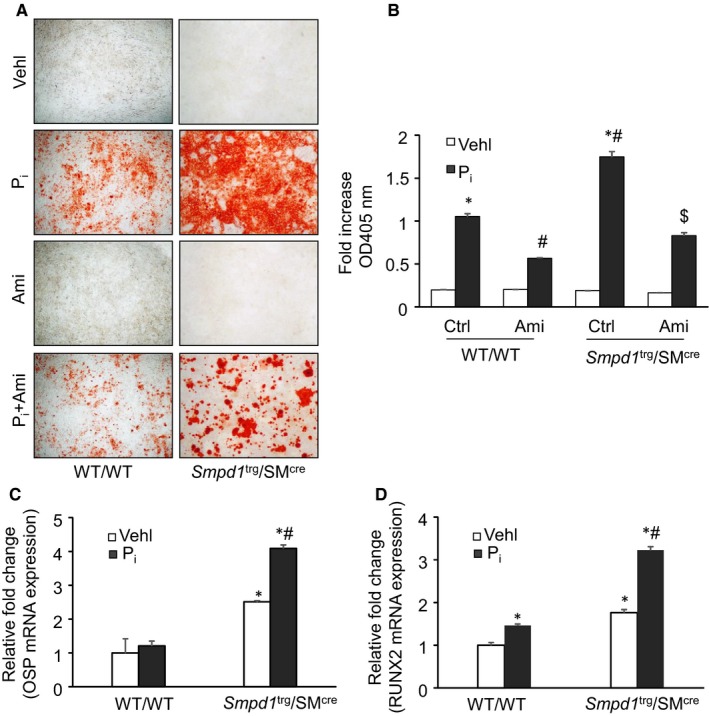
Effect of SMC‐specific *Smpd1* overexpression on P_i_‐induced calcification and phenotype change in SMC‐specific *Smpd1* transgenic CASMCs in vitro*.* A, Representative images showed increased calcium deposition in P_i_‐treated *Smpd1* ^trg^/SM^cre^ CASMCs. B, Summarized bar graph showed significant increased P_i_‐induced mineralization in *Smpd1* ^trg^/SM^cre^, which was significantly decreased by amitriptyline (Ami). Summarized bar graph showed mRNA expression of C. OSP and D. RUNX2 in P_i_‐treated CASMCs by RT‐PCR. n = 3. Ami, amitriptyline; OSP, osteopontin; P_i_, high phosphate; RUNX2, runt‐related transcription factor 2; SMC, smooth muscle cell; Vehl, vehicle. *P ˂ .05 vs WT/WT Vehl; #*P* ˂ .05 vs WT/WT P_i_; $*P* ˂ .05 vs* Smpd1* ^trg^/SM^cre^ P_i_ by two‐way ANOVA followed by Duncan's test

In our in vitro study, using CASMCs, we determined the effect of Cre‐mediated overexpression of *Smpd1* gene in high phosphate (P_i_)‐induced calcification model. As shown in Figure 7A,B, calcium deposition as shown by the presence of Alizarin Red‐stained nodules was significantly increased with P_i_ treatment in CASMCs isolated from *Smpd1* ^trg^/SM^cre^ mice as compared to WT/WT cells, which were significantly decreased by ASM inhibition by amitriptyline.

Using real‐time PCR, we observed that SMC‐specific overexpression of *Smpd1* gene induced the osteogenic phenotypic conversion in P_i_‐treated CASMCs, as depicted by significantly increased expression of OSP and RUNX2 (Figure 7C,D). Together, these results indicate that lysosomal ceramide‐sphingolipid contributes to the phenotypic transition during the development of calcification.

### 3.6 Lysosome‐MVB interactions and sEV secretion in the P_i_ *‐*treated* Smpd1* ^trg^/SM^cre^ mice CASMCs in vitro

**Figure 8:**
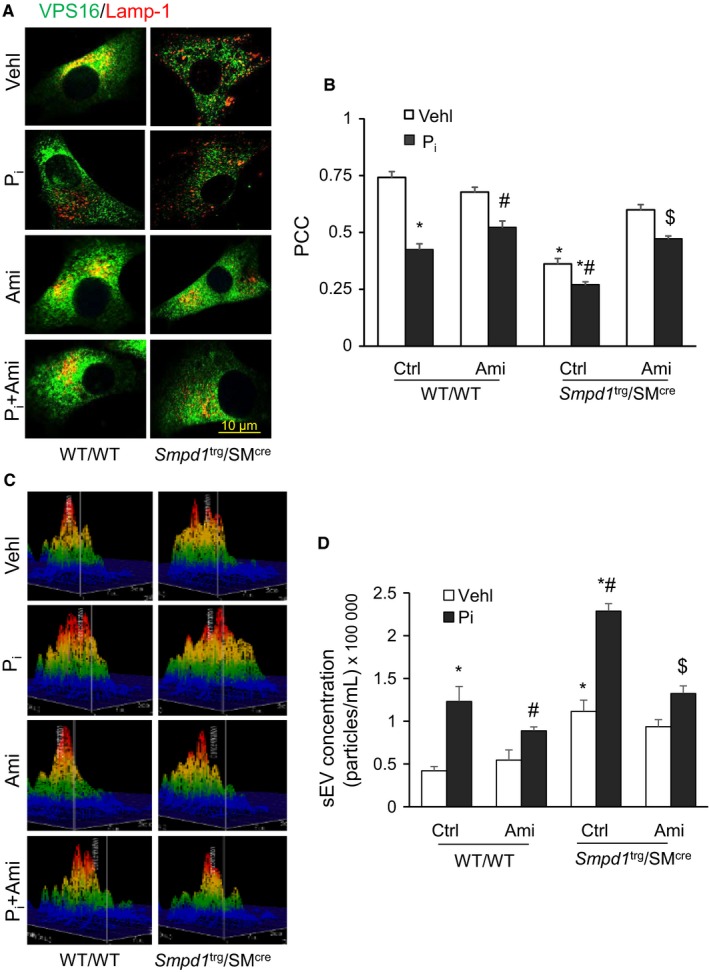
Lysosome‐MVB interactions and sEV excretion in the P_i_‐treated SMC‐specific *Smpd1* transgenic CASMCs in vitro*.* A, Representative confocal images showed co‐localization of VPS16 (green) and Lamp‐1(red) in CASMCs B. Bar graph shows significant decrease in co‐localization of VPS16/Lamp‐1 in P_i_‐treated *Smpd1* ^trg^/SM^cre^ CASMCs, which was significantly increased by Ami. n = 3. C, High P_i_ treatment increased sEV release from *Smpd1* ^trg^/SM^cre^ CASMCs. D, Bar graph shows significantly increased sEV release from CASMCs of SMC‐specific *Smpd1* transgenic mice, which was significantly decreased through ASM inhibition by Ami, n = 3. Ami, amitriptyline; PCC, Pearson correlation coefficient; P_i_, high phosphate; SMC, smooth muscle cell; Vehl, vehicle. *P ˂ .05 vs WT/WT Vehl; #*P* ˂ .05 vs WT/WT P_i_; $*P *˂ .05 vs* Smpd1* ^trg^/SM^cre^ P_i_. Data are shown as mean ± SEM of values

Using confocal microscopy, we observed increased co‐localization of VPS16 (MVB marker, green) and Lamp‐1 (lysosome marker, red), indicating MVB interactions or even fusion with lysosomes in WT/WT CASMCs. In the CASMCs without P_i_ treatment, overexpression of *Smpd1* gene had reduced co‐localization of VPS16 vs Lamp‐1 (fewer yellows dots) as compared to WT/WT cells, while as P_i_ exposure decreased co‐localization of both the markers in CASMCs from WT/WT as well as *Smpd1* ^trg^/SM^cre^ (Figure 8A). However, amitriptyline treatment significantly increased the co‐localization of VPS16 vs Lamp‐1 (larger yellow spots) in P_i_‐treated CASMCs both in *Smpd1* ^trg^/SM^cre^ and WT/WT cells. The bar graphs represent the co‐localization coefficient (PPC), exhibiting decreased interaction of lysosomes and MVBs more in P_i_‐treated *Smpd1* ^trg^/SM^cre^ CASMCs as compared to WT/WT cells, which was significantly increased by amitriptyline (Figure 8B).

Quantification of sEVs using a nanoparticle tracking analysis system showed that P_i_ treatment in CASMCs significantly increased secretion of sEVs (<200 nm), as shown by representative 3‐D histograms in Figure 8C and more particles in <200 nm in P_i_‐treated CASMCs from SMC‐specific *Smpd1* transgenic mice, while as amitriptyline significantly decreased P_i_‐induced sEV secretion. A bar graph shows vesicle counts of <200 nm size (Figure 8D). These data confirm that sEV release increased from SMCs with overexpression of *Smpd1* gene even without stimulation by P_i_, which may drive arterial calcification.

### 3.7 Overexpression of SMC‐specific *Smpd1 *accelerates arterial stiffness in *Smpd1* transgenic mice

**Figure 9:**
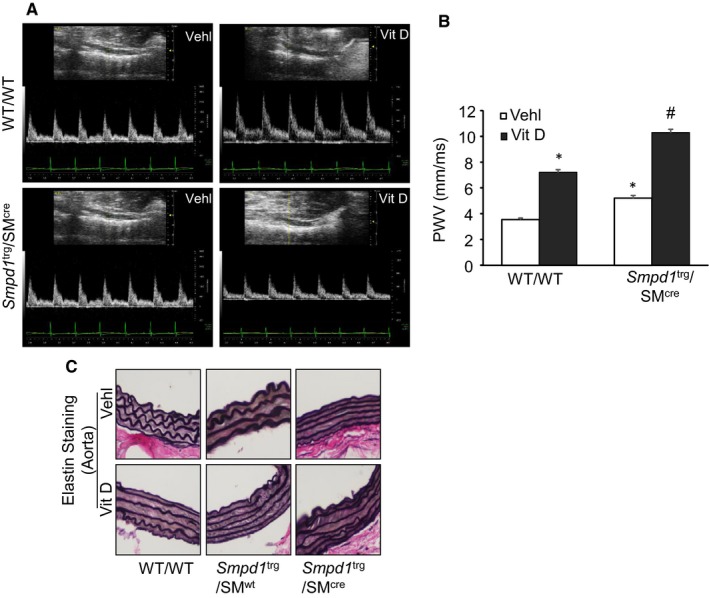
Effect of SMC‐specific *Smpd1* overexpression on arterial stiffness in Vit D‐treated SMC‐specific *Smpd1* transgenic mice. A, Representative Doppler images showed Doppler velocity signals. B, Bar graph shows significant increase in PWV of *Smpd1* ^trg^/SM^cre^ as compared to control WT/WT littermates. C, Representative images of aortic sections showed damage to elastic fibres, distorted junctions of the innermost and outermost layers, and formation of solid plates or sheaths. n = 5. SMC: smooth muscle cell; Vehl: vehicle; Vit D: vitamin D. *P ˂ .05 vs WT/WT Vehl; #*P* ˂ .05 vs WT/WT Vit D group. Data are shown as mean ± SEM of values

Pulse wave velocity directly correlates with arterial stiffness and inversely proportional to arterial distensibility.^48^ As shown in Figure 9A,B, SMC‐specific overexpression of *Smpd1* significantly increased PWV as compared to control WT/WT littermates, suggesting that aortic wall stiffening and remodelling in *Smpd1* ^trg^/SM^cre^ mice even before frank aortic medial calcification can be observed. Furthermore, PWV was significantly increased both in WT/WT and *Smpd1* ^trg^/SM^cre^ mice under Vit D‐treated conditions. However, PWV was significantly increased in Vit D‐treated *Smpd1* ^trg^/SM^cre^ mice as compared to Vit D‐treated WT/WT mice, confirming that increased lysosomal ceramide due to overexpression of *Smpd1* gene plays a role in arterial stiffness.

Further, we performed the elastin staining to determine whether elastin degradation occurred in this animal model. WT/WT and *Smpd1* ^trg^/SM^wt^ control aortas showed intact elastic lamellae (Figure 9C) while as upon receiving Vit D treatment, elastic lamellae appeared to be disorganized. In control *Smpd1* ^trg^/SM^cre^ mice, integrity of the elastic laminae in the media of aortas seems to be compromised, and with Vit D treatment thinning of elastin, distorted junctions of the innermost and outermost layers, and formation of solid plates or sheaths disorganization were readily observed in the calcified aortas of *Smpd1* ^trg^/SM^cre^ mice as shown in Figure 9C.

## 4 DISCUSSION

In the present study, we describe for the first time that lysosomal ceramide plays a critical role in sEV release, phenotype transition and mineral deposition in arterial SMCs leading to AMC. Overexpression of *Smpd1* gene that encodes a lysosomal ASM to increase ceramide showed that lysosomal sphingolipid/ceramide pathway in SMCs may be a contributing factor that determine the development of AMC. Using a non‐invasive imaging technique, we found that overexpression of *Smpd1* in SMCs of *Smpd1* ^trg^/SM^cre^ mice resulted in increased arterial stiffness as compared to their littermates. Moreover, overexpression of *Smpd1* gene in SMCs using *Smpd1* ^trg^/SM^cre^ mice largely enhanced calcification in the aortic medial and coronary arterial wall than their littermates during hypercalcaemia induced by high doses of Vit D. However, using amitriptyline, a pharmacological inhibitor of ASM significantly reduced both aortic and coronary arterial wall calcification.

Our histopathological studies showed that in *Smpd1* ^trg^/SM^cre^ mice, enhanced damage to elastic fibres and distorted junctions of the innermost and outermost layers occurred with the formation of solid plates or sheaths, which in turn damaged the architecture of aortic medial wall. We also observed decreased co‐localization of MVBs (VPS16) and lysosome (Lamp‐1) in aortic medial wall while as increased expression of sEV markers such as CD63, AnX2 and ALP were observed in coronary arterial wall in Vit D‐treated *Smpd1* ^trg^/SM^cre^ mice compared with their littermates (*Smpd1* ^trg^/SM^wt^ and controls). In addition, SMC lineage marker SM22‐α was significantly decreased, while as ‘bone’ transcription factors (eg RUNX2) and matrix proteins (eg osteopontin (OSP)) remarkably increased in the arterial wall in Vit D‐treated *Smpd1* ^trg^/SM^cre^ mice compared with their littermates (*Smpd1* ^trg^/SM^wt^ and controls), which was prevented by amitriptyline. Furthermore, under in vitro conditions in CASMCs, we observed that Cre‐mediated overexpression of *Smpd1* gene increased calcium deposition, sEV secretion, increased OSP and RUNX2 expression associated with decreased co‐localization of VPS16 and Lamp‐1 in P_i_‐induced calcification model. Conversely, inhibition of ASM by amitriptyline decreased sEV secretion and calcium deposition in CASMCs in vitro. These pathological changes in AMC development and SMC phenotypic transition due to specific overexpression of *Smpd1* gene in SMCs were demonstrated to be mainly associated with increased ceramide in lysosome of these cells because the SMC‐specific *Smpd1* transgenic mice (*Smpd1* ^trg^/SM^cre^) caused similar changes in aortic and coronary arterial wall. To our knowledge, these findings provide evidence that lysosome ceramide/sphingolipid metabolism may be critically involved in the development of AMC.

Overexpression of *Smpd1* in SMCs results in increased production of ceramide from sphingomyelin in *Smpd1* ^trg^/SM^cre^ mice.^49^ Although there is no direct evidence regarding the role of lysosome ceramide in AMC, some studies provided scientific premise for our studies in this area. For example, Song et al^50^ demonstrated that TLR4 regulates vascular calcificafication in VSMCs and  C2‐ceramide treatment rescued vascular calcification inhibited by pyrrolidine dithiocarbamate (PDTC) suggesting that TLR4/NF‐κB/Ceramide signalling mediates Ox‐LDL‐induced calcification of human VSMCs. It has been reported that the imbalanced mineral metabolism in SMCs may increase sEV release via sphingolipid/ceramide pathway, which triggers more secretion of calcifying exosomes, resulting in arterial calcification.^10^ Also, increased ceramide production in cell membrane or cytoplasm via SMPD3 pathway may induce sEV biogenesis leading to arterial calcification,^10^, ^47^ which represents a different mechanism from lysosomal regulation of sEV secretion shown in the present study. Vascular SMC‐derived vesicles were enriched with sEV markers such as CD63, AnX2 and ALP, and their production and fetuin‐A recycling are regulated by SMPD3, a known regulator of exosome biogenesis.^47^ We found that SMC‐specific overexpression of *Smpd1* gene substantially reduces lysosome‐MVB interactions in aortic medial SMCs (VPS16 vs Lamp‐1), which may decrease lysosome degradation of MVBs, increasing the fusion of MVBs with the plasma membrane to release sEVs. Increased exosomes or sEV in arterial interstitial spaces may initiate nidus for mineralization.^8^, ^10^ Furthermore, we observed that sEV markers, CD63, AnX2 and ALP, were increased in the coronary arterial wall of SMC‐specific *Smpd1* transgenic mice, and have increased ceramide locally in medial SMCs. However, inhibition of ASM pharmacologically by amitriptyline decreased lysosome fusion with MVBs, observed by decreased VPS16 and Lamp‐1 co‐localization, also reduced the expression of sEV markers such as CD63, AnX2 and ALP. Recent studies have indicated that intimal and medial vascular calcification may be mediated by a common mechanism, namely the large increases in extracellular vesicles in the vascular interstitial space, in particular, the sEV_S_ or exosomes (with size of 40‐100 or to 140 nm). These sEVs are mainly produced and excreted from arterial SMCs.^8^, ^10^, ^51^ Co‐culture of matrix vesicles (MVs) isolated from vascular SMCs of rats having chronic kidney disease with SMCs from normal littermates demonstrated that endocytosis of these MVs results in increased [Ca^2+^]_i_ of recipient normal vascular SMCs and accelerated calcification.^52^ However, the mechanisms mediating sEV biogenesis and secretion in SMCs remain poorly understood. It has been observed that ASM is a key enzyme involved microvesicle biogenesis in glial cells (microglia and astrocytes) via activation of P38 MAP kinase where it catalyses CER formation from sphingomyelin.^24^ In the oxidized LDL‐activated human macrophage cell line, U937, it was found that activation of ASM contributes to the release of IL‐1β in association with exosomes.^26^ Also, it was found that pharmacological (desipramine) or genetical (ASM siRNA) inhibition of ASM reduced exosome and IL‐1β secretion. Cigarette smoke (CS)‐induced activation of ASM increased extracellular vesicle (EV) production in lung endothelial cells while as its inhibition with imipramine decreased CS‐induced EV production.^27^ These in vitro findings were validated in ASM (*Smpd1* ^−/−^) knockout mice which showed decreased levels of EVs in plasma, while as its overexpression increased circulating EVs. Another study reported that CS promoted microvesicle shedding via activation of p38 MAPK mediated by an ASM‐dependent pathway.^24^, ^28^ By nanoparticle tracking analysis, we demonstrated that sEV secretion was increased in CASMCs from *Smpd1* transgenic mice which were decreased by amitriptyline. PDMP, a ceramide analogue, reduced osteoblastic proliferation and pre‐osteoblastic cell differentiation by translocation of mTORC1 from late endosome/Lysosome to the endoplasmic reticulum, suggesting inhibition of mTORC1 activity. This inhibitory action of ceramide analogue allows normal lysosomal functioning or lysosomal activation and their fusion with MVBs.^33^ Together, all these studies conclude that activation of ASM promotes sEV release under various pathological conditions.

The phenotype change in SMCs shares some features with mineralizing SMC phenotype, including loss of smooth muscle lineage markers and up‐regulation of osteopontin.^53^ In AMC, osteogenic phenotype can be observed in vascular medial cells both in humans and experimental animals, and these cells are known as major mediators of vascular calcification.^54^, ^55^, ^56^ In this context, in human femoral arterial SMCs it was observed that Ox‐LDL‐induced matrix mineralization may be mediated by ceramide,^57^ which was due to increased neutral sphingomyelinase activity and ceramide levels. GW4869, a neutral sphingomyelinase (N‐SMase) inhibitor, significantly reduced Ox‐LDL‐induced calcification in these cultured SMCs.^57^

In the present study, we found that the overexpression of *Smpd1* gene has little effect on the basal expression of SMC marker gene SM22‐α. In contrast, the impact of the overexpression of *Smpd1* gene on Vit D‐induced expression of SMC marker was dramatic, as shown by the significant decrease in the expression of SM22‐α and increased OSP and RUNX2. However, treatment with amitriptyline, a functional inhibitor of ASM, markedly increased the expression of SM22‐α whereas decreased OSP and RUNX2 expression was observed. Also, mRNA expression of OSP and RUNX2 was increased both in ASM overexpressed vehicle and in P_i_‐treated CASMCs. These findings confirm the idea that lysosomal *Smpd1* expression‐associated sphingolipid‐ceramide pathway in SMCs may be one of the contributing factors for the phenotype change in SMCs undergoing AMC.

Medial calcification increases arterial stiffness that develop along the concentric elastin lamellae during ageing, detected as continuous linear hydroxyapatite deposits in the absence of inflammatory cells.^58^, ^59^ Cellular sphingolipids alterations appear to be a hallmark of ageing; in particular, ceramide could contribute to age‐related remodelling of the vasculature. Various studies reported that ceramide promotes collagen deposition, lung and liver fibrosis.^60^, ^61^ Moreover, ceramide is known to regulate actin cytoskeleton dynamics and its alteration has been reported in VSMCs from large arteries with age.^62^, ^63^ Actin cytoskeleton regulation is an important component of VSMC mechanosensing^64^ and the myogenic response,^65^ suggesting that ceramide could also play a role in impaired mechano‐transduction in small arteries with ageing. In the current study, we tried to investigate whether the increased lysosomal ceramide in SMCs contributes to arterial plasticity by measuring PWV as an index of arterial stiffness.^66^ We found that SMC‐specific overexpression of *Smpd1* significantly increased PWV suggesting that increased lysosomal ceramide due to overexpression of *Smpd1* in SMCs contributed to the arterial stiffness during the development of AMC. In this context, study in ApoE^−/−^ mice and rabbits fed a Western diet reported that inhibition of glycosphingolipid synthesis can prevent the development of atherosclerosis and lower arterial stiffness independent of blood pressure.^30^ Hence, these studies provide evidence that sphingolipid biosynthesis pathway could be a target for the prevention of arterial stiffness during AMC.

In summary, this study demonstrates remodelling of arteries during AMC that is accompanied by lysosomal enzyme ASM controlling ceramide production in arterial SMCs. Lysosomal overexpression of *Smpd1* gene specifically in SMCs may be crucially involved in the secretion of sEVs and phenotypic switch in arterial SMCs, initiating AMC. This suggests sphingolipids may be important mediators of vascular calcification. Given that arterial medial calcification is a major risk factor for cardiovascular disease, our study opens a new area for further research into the mechanisms that underlie vascular remodelling in AMC.

## CONFLICT OF INTEREST

The authors declare no conflict interests.

## AUTHOR CONTRIBUTIONS

OMB and PL planned and designed the studies. F.S provided technical facility for various experiments. XY characterized and maintained mice. OMB and C.C conducted experiments and generated data. OMB analysed and interpreted data. OMB and PL wrote and revised the manuscript. All authors approved the final version of the manuscript.

## Data Availability

All data generated or analysed during this study are included in this article.

## References

[1] Shanahan CM , Crouthamel MH , Kapustin A , Giachelli CM . Arterial calcification in chronic kidney disease: key roles for calcium and phosphate. Circ Res. 2011;109:697‐711. 21885837 10.1161/CIRCRESAHA.110.234914 PMC3249146

[2] Reynolds JL , Joannides AJ , Skepper JN , et al. Human vascular smooth muscle cells undergo vesicle‐mediated calcification in response to changes in extracellular calcium and phosphate concentrations: a potential mechanism for accelerated vascular calcification in ESRD. J Am Soc Nephrol. 2004;15:2857‐2867. 15504939 10.1097/01.ASN.0000141960.01035.28

[3] Tanimura A , McGregor DH , Anderson HC . Matrix vesicles in atherosclerotic calcification. Proc Soc Exp Biol Med. 1983;172:173‐177. 6828462 10.3181/00379727-172-41542

[4] Shroff RC , McNair R , Skepper JN , et al. Chronic mineral dysregulation promotes vascular smooth muscle cell adaptation and extracellular matrix calcification. J Am Soc Nephrol. 2010;21:103‐112. 19959717 10.1681/ASN.2009060640 PMC2799273

[5] Shroff RC , McNair R , Figg N , et al. Dialysis accelerates medial vascular calcification in part by triggering smooth muscle cell apoptosis. Circulation. 2008;118:1748‐1757. 18838561 10.1161/CIRCULATIONAHA.108.783738

[6] El‐Abbadi MM , Pai AS , Leaf EM , et al. Phosphate feeding induces arterial medial calcification in uremic mice: role of serum phosphorus, fibroblast growth factor‐23, and osteopontin. Kidney Int. 2009;75:1297‐1307. 19322138 10.1038/ki.2009.83 PMC2799244

[7] Speer MY , Yang HY , Brabb T , et al. Smooth muscle cells give rise to osteochondrogenic precursors and chondrocytes in calcifying arteries. Circ Res. 2009;104:733‐741. 19197075 10.1161/CIRCRESAHA.108.183053 PMC2716055

[8] Kapustin AN , Shanahan CM . Emerging roles for vascular smooth muscle cell exosomes in calcification and coagulation. J Physiol. 2016;594:2905‐2914. 26864864 10.1113/JP271340 PMC4887700

[9] Tyson KL , Reynolds JL , McNair R , Zhang Q , Weissberg PL , Shanahan CM . Osteo/chondrocytic transcription factors and their target genes exhibit distinct patterns of expression in human arterial calcification. Arterioscler Thromb Vasc Biol. 2003;23:489‐494. 12615658 10.1161/01.ATV.0000059406.92165.31

[10] Kapustin AN , Chatrou ML , Drozdov I , et al. Vascular smooth muscle cell calcification is mediated by regulated exosome secretion. Circ Res. 2015;116:1312‐1323. 25711438 10.1161/CIRCRESAHA.116.305012

[11] Giussani P , Tringali C , Riboni L , Viani P , Venerando B . Sphingolipids: key regulators of apoptosis and pivotal players in cancer drug resistance. Int J Mol Sci. 2014;15:4356‐4392. 24625663 10.3390/ijms15034356 PMC3975402

[12] Hannun YA , Obeid LM . Principles of bioactive lipid signalling: lessons from sphingolipids. Nat Rev Mol Cell Biol. 2008;9:139‐150. 18216770 10.1038/nrm2329

[13] Chiricozzi E , Loberto N , Schiumarini D , et al. Sphingolipids role in the regulation of inflammatory response: from leukocyte biology to bacterial infection. J Leukoc Biol. 2018;103:445‐456. 29345379 10.1002/JLB.3MR0717-269R

[14] Airola MV , Hannun YA . Sphingolipid metabolism and neutral sphingomyelinases In: Handbook of Experimental Pharmacology. 2013;(215)57‐76. 10.1007/978-3-7091-1368-4_3 PMC4043343 23579449

[15] Ogretmen B . Sphingolipid metabolism in cancer signalling and therapy. Nat Rev Cancer. 2018;18:33‐50. 29147025 10.1038/nrc.2017.96 PMC5818153

[16] Zhou Y , Salker MS , Walker B , et al. Acid Sphingomyelinase (ASM) is a negative regulator of regulatory T cell (Treg) development. Cell Physiol Biochem. 2016;39:985‐995. 27512981 10.1159/000447806

[17] Boini KM , Xia M , Abais JM , Xu M , Li CX , Li PL . Acid sphingomyelinase gene knockout ameliorates hyperhomocysteinemic glomerular injury in mice lacking cystathionine‐beta‐synthase. PLoS ONE. 2012;7:e45020. 23024785 10.1371/journal.pone.0045020 PMC3443210

[18] Boini KM , Xia M , Li C , et al. Acid sphingomyelinase gene deficiency ameliorates the hyperhomocysteinemia‐induced glomerular injury in mice. Am J Pathol. 2011;179:2210‐2219. 21893018 10.1016/j.ajpath.2011.07.019 PMC3204029

[19] Boini KM , Xia M , Koka S , Gehr TW , Li PL . Instigation of NLRP3 inflammasome activation and glomerular injury in mice on the high fat diet: role of acid sphingomyelinase gene. Oncotarget. 2016;7:19031‐19044. 26980705 10.18632/oncotarget.8023 PMC4951349

[20] Koka S , Xia M , Chen Y , et al. Endothelial NLRP3 inflammasome activation and arterial neointima formation associated with acid sphingomyelinase during hypercholesterolemia. Redox Biol. 2017;13:336‐344. 28633109 10.1016/j.redox.2017.06.004 PMC5479959

[21] Fowler S . Lysosomal localization of sphingomyelinase in rat liver. Biochem Biophys Acta. 1969;191:481‐484. 4311152 10.1016/0005-2744(69)90271-x

[22] Perrotta C , Bizzozero L , Cazzato D , et al. Syntaxin 4 is required for acid sphingomyelinase activity and apoptotic function. J Biol Chem. 2010;285:40240‐40251. 20956541 10.1074/jbc.M110.139287 PMC3001005

[23] Grassme H , Jekle A , Riehle A , et al. CD95 signaling via ceramide‐rich membrane rafts. J Biol Chem. 2001;276:20589‐20596. 11279185 10.1074/jbc.M101207200

[24] Bianco F , Perrotta C , Novellino L , et al. Acid sphingomyelinase activity triggers microparticle release from glial cells. EMBO J. 2009;28:1043‐1054. 19300439 10.1038/emboj.2009.45 PMC2664656

[25] Subra C , Laulagnier K , Perret B , Record M . Exosome lipidomics unravels lipid sorting at the level of multivesicular bodies. Biochimie. 2007;89:205‐212. 17157973 10.1016/j.biochi.2006.10.014

[26] Truman JP , Al Gadban MM , Smith KJ , et al. Differential regulation of acid sphingomyelinase in macrophages stimulated with oxidized low‐density lipoprotein (LDL) and oxidized LDL immune complexes: role in phagocytosis and cytokine release. Immunology. 2012;136:30‐45. 22236141 10.1111/j.1365-2567.2012.03552.x PMC3372755

[27] Serban KA , Rezania S , Petrusca DN , et al. Structural and functional characterization of endothelial microparticles released by cigarette smoke. Sci Rep. 2016;6:31596. 27530098 10.1038/srep31596 PMC4987682

[28] Li CJ , Liu Y , Chen Y , Yu D , Williams KJ , Liu ML . Novel proteolytic microvesicles released from human macrophages after exposure to tobacco smoke. Am J Pathol. 2013;182:1552‐1562. 23499464 10.1016/j.ajpath.2013.01.035 PMC3644720

[29] Heinrich M , Wickel M , Schneider‐Brachert W , et al. Cathepsin D targeted by acid sphingomyelinase‐derived ceramide. EMBO J. 1999;18:5252‐5263. 10508159 10.1093/emboj/18.19.5252 PMC1171596

[30] Chatterjee S , Bedja D , Mishra S , et al. Inhibition of glycosphingolipid synthesis ameliorates atherosclerosis and arterial stiffness in apolipoprotein E‐/‐ mice and rabbits fed a high‐fat and ‐cholesterol diet. Circulation. 2014;129:2403‐2413. 24710030 10.1161/CIRCULATIONAHA.113.007559 PMC4053506

[31] Mishra S , Bedja D , Amuzie C , et al. Improved intervention of atherosclerosis and cardiac hypertrophy through biodegradable polymer‐encapsulated delivery of glycosphingolipid inhibitor. Biomaterials. 2015;64:125‐135. 26111596 10.1016/j.biomaterials.2015.06.001 PMC4557963

[32] Fukumoto S , Iwamoto T , Sakai E , et al. Current topics in pharmacological research on bone metabolism: osteoclast differentiation regulated by glycosphingolipids. J Pharmacol Sci. 2006;100:195‐200. 16538029 10.1254/jphs.fmj05004x3

[33] Ode T , Podyma‐Inoue KA , Terasawa K , et al. PDMP, a ceramide analogue, acts as an inhibitor of mTORC1 by inducing its translocation from lysosome to endoplasmic reticulum. Exp Cell Res. 2017;350:103‐114. 27865938 10.1016/j.yexcr.2016.11.011

[34] Li X , Xu M , Pitzer AL , et al. Control of autophagy maturation by acid sphingomyelinase in mouse coronary arterial smooth muscle cells: protective role in atherosclerosis. J Mol Med (Berl). 2014;92:473‐485. 24463558 10.1007/s00109-014-1120-y PMC4211081

[35] Rangrez AY , M'Baya‐Moutoula E , Metzinger‐Le Meuth V , et al. Inorganic phosphate accelerates the migration of vascular smooth muscle cells: evidence for the involvement of miR‐223. PLoS ONE. 2012;7:e47807. 23094093 10.1371/journal.pone.0047807 PMC3475714

[36] Zhu D , Mackenzie NC , Shanahan CM , Shroff RC , Farquharson C , MacRae VE . BMP‐9 regulates the osteoblastic differentiation and calcification of vascular smooth muscle cells through an ALK1 mediated pathway. J Cell Mol Med. 2015;19:165‐174. 25297851 10.1111/jcmm.12373 PMC4288360

[37] Hong J , Bhat OM , Li G , et al. Lysosomal regulation of extracellular vesicle excretion during d‐ribose‐induced NLRP3 inflammasome activation in podocytes. Biochim Biophys Acta. 2019;1866:849‐860. 10.1016/j.bbamcr.2019.02.007 PMC6800119 30771382

[38] Yuan X , Bhat OM , Lohner H , Li N , Zhang Y , Li PL . Inhibitory effects of growth differentiation factor 11 on autophagy deficiency‐induced dedifferentiation of arterial smooth muscle cells. Am J Physiol Heart Circ Physiol. 2019;316:H345‐H356. 30462553 10.1152/ajpheart.00342.2018 PMC6397385

[39] Sokolova V , Ludwig AK , Hornung S , et al. Characterisation of exosomes derived from human cells by nanoparticle tracking analysis and scanning electron microscopy. Colloids Surf, B. 2011;87:146‐150. 10.1016/j.colsurfb.2011.05.013 21640565

[40] Price PA , Buckley JR , Williamson MK . The amino bisphosphonate ibandronate prevents vitamin D toxicity and inhibits vitamin D‐induced calcification of arteries, cartilage, lungs and kidneys in rats. J Nutrition. 2001;131:2910‐2915. 11694617 10.1093/jn/131.11.2910

[41] Peng H , Li C , Kadow S , et al. Acid sphingomyelinase inhibition protects mice from lung edema and lethal Staphylococcus aureus sepsis. J Mol Med (Berl). 2015;93:675‐689. 25616357 10.1007/s00109-014-1246-y PMC4432103

[42] Hoenderop JG , van der Kemp AW , Urben CM , Strugnell SA , Bindels RJ . Effects of vitamin D compounds on renal and intestinal Ca2+ transport proteins in 25‐hydroxyvitamin D3–1alpha‐hydroxylase knockout mice. Kidney Int. 2004;66:1082‐1089. 15327402 10.1111/j.1523-1755.2004.00858.x

[43] Bhat OM , Kumar PU , Giridharan NV , Kaul D , Kumar MJ , Dhawan V . Interleukin‐18‐induced atherosclerosis involves CD36 and NF‐kappaB crosstalk in Apo E‐/‐ mice. J Cardiol. 2015;66:28‐35. 25475966 10.1016/j.jjcc.2014.10.012

[44] Yuan X , Bhat OM , Meng N , Lohner H , Li PL . Protective role of autophagy in Nlrp3 inflammasome activation and medial thickening of mouse coronary arteries. Am J Pathol. 2018;188:2948‐2959. 30273598 10.1016/j.ajpath.2018.08.014 PMC6334256

[45] Tong X , Khandelwal AR , Wu X , et al. Pro‐atherogenic role of smooth muscle Nox4‐based NADPH oxidase. J Mol Cell Cardiol. 2016;92:30‐40. 26812119 10.1016/j.yjmcc.2016.01.020 PMC5008453

[46] Beckmann N , Kadow S , Schumacher F , et al. Pathological manifestations of Farber disease in a new mouse model. Biol Chem. 2018;399:1183‐1202. 29908121 10.1515/hsz-2018-0170

[47] Trajkovic K , Hsu C , Chiantia S , et al. Ceramide triggers budding of exosome vesicles into multivesicular endosomes. Science. 2008;319:1244‐1247. 18309083 10.1126/science.1153124

[48] Lane HA , Smith JC , Davies JS . Noninvasive assessment of preclinical atherosclerosis. Vasc Health Risk Manag. 2006;2:19‐30. 17319466 10.2147/vhrm.2006.2.1.19 PMC1993970

[49] Bhat OM , Yuan X , Li G , Lee R , Li PL . Sphingolipids and redox signaling in renal regulation and chronic kidney diseases. Antioxid Redox Signal. 2018;28:1008‐1026 10.1089/ars.2017.7129 PMC5849286 29121774

[50] Song Y , Hou M , Li Z , et al. TLR4/NF‐kappaB/Ceramide signaling contributes to Ox‐LDL‐induced calcification of human vascular smooth muscle cells. Eur J Pharmacol. 2017;794:45‐51. 27876618 10.1016/j.ejphar.2016.11.029

[51] Kapustin AN , Schoppet M , Schurgers LJ , et al. Prothrombin Loading of Vascular Smooth Muscle Cell‐Derived Exosomes Regulates Coagulation and Calcification. Arterioscler Thromb Vasc Biol. 2017;37:e22‐e32. 28104608 10.1161/ATVBAHA.116.308886

[52] Chen NX , O'Neill KD , Moe SM . Matrix vesicles induce calcification of recipient vascular smooth muscle cells through multiple signaling pathways. Kidney Int. 2018;93:343‐354. 29032812 10.1016/j.kint.2017.07.019 PMC8211355

[53] Giachelli CM , Liaw L , Murry CE , Schwartz SM , Almeida M . Osteopontin expression in cardiovascular diseases. Ann N Y Acad Sci. 1995;760:109‐126. 7785890 10.1111/j.1749-6632.1995.tb44624.x

[54] Krohn JB , Hutcheson JD , Martinez‐Martinez E , et al. Discoidin domain receptor‐1 regulates calcific extracellular vesicle release in vascular smooth muscle cell fibrocalcific response via transforming growth factor‐beta signaling. Arterioscler Thromb Vasc Biol. 2016;36:525‐533. 26800565 10.1161/ATVBAHA.115.307009 PMC4767541

[55] Pai A , Leaf EM , El‐Abbadi M , Giachelli CM . Elastin degradation and vascular smooth muscle cell phenotype change precede cell loss and arterial medial calcification in a uremic mouse model of chronic kidney disease. Am J Pathol. 2011;178:764‐773. 21281809 10.1016/j.ajpath.2010.10.006 PMC3069837

[56] Zebger‐Gong H , Muller D , Diercke M , et al. 1,25‐Dihydroxyvitamin D3‐induced aortic calcifications in experimental uremia: up‐regulation of osteoblast markers, calcium‐transporting proteins and osterix. J Hypertens. 2011;29:339‐348. 21063202 10.1097/HJH.0b013e328340aa30

[57] Liao L , Zhou Q , Song Y , et al. Ceramide mediates Ox‐LDL‐induced human vascular smooth muscle cell calcification via p38 mitogen‐activated protein kinase signaling. PLoS ONE. 2013;8:e82379. 24358176 10.1371/journal.pone.0082379 PMC3865066

[58] Shroff RC , Shanahan CM . The vascular biology of calcification. Semin Dial. 2007;20:103‐109. 17374082 10.1111/j.1525-139X.2007.00255.x

[59] Mackey RH , Venkitachalam L , Sutton‐Tyrrell K . Calcifications, arterial stiffness and atherosclerosis. Adv Cardiol. 2007;44:234‐244. 17075212 10.1159/000096744

[60] Sato M , Markiewicz M , Yamanaka M , et al. Modulation of transforming growth factor‐beta (TGF‐beta) signaling by endogenous sphingolipid mediators. J Biol Chem. 2003;278:9276‐9282. 12515830 10.1074/jbc.M211529200

[61] Dhami R , He X , Schuchman EH . Acid sphingomyelinase deficiency attenuates bleomycin‐induced lung inflammation and fibrosis in mice. Cell Physiol Biochem. 2010;26:749‐760. 21063112 10.1159/000322342 PMC3048941

[62] Qiu H , Zhu Y , Sun Z , et al. Short communication: vascular smooth muscle cell stiffness as a mechanism for increased aortic stiffness with aging. Circ Res. 2010;107:615‐619. 20634486 10.1161/CIRCRESAHA.110.221846 PMC2936100

[63] Zeidan YH , Jenkins RW , Hannun YA . Remodeling of cellular cytoskeleton by the acid sphingomyelinase/ceramide pathway. J Cell Biol. 2008;181:335‐350. 18426979 10.1083/jcb.200705060 PMC2315679

[64] Hill MA , Meininger GA . Arteriolar vascular smooth muscle cells: mechanotransducers in a complex environment. Int J Biochem Cell Biol. 2012;44:1505‐1510. 22677491 10.1016/j.biocel.2012.05.021 PMC4221252

[65] Walsh MP , Cole WC . The role of actin filament dynamics in the myogenic response of cerebral resistance arteries. J Cereb Blood Flow Metab. 2013;33:1‐12. 23072746 10.1038/jcbfm.2012.144 PMC3597360

[66] Hartley CJ , Taffet GE , Michael LH , Pham TT , Entman ML . Noninvasive determination of pulse‐wave velocity in mice. Am J Physiol. 1997;273:H494‐500. 9249523 10.1152/ajpheart.1997.273.1.H494

