# Supplementary material for: Medial calcification in the arterial wall of smooth muscle cell‐specific Smpd1 transgenic mice: A ceramide‐mediated vasculopathy
Source: J Cell Mol Med. 2019 Nov 19;24(1):539–53. doi: 10.1111/jcmm.14761 (PMC6933411; doi:10.1111/jcmm.14761)
Supplement: Supplementary file 1 [file JCMM-24-539-s001.pdf]

Medial Calcification in the arterial wall of Smooth Muscle Cell-Specific *Smpd1* Transgenic Mice: A Ceramide-Mediated Vasculopathy

Owais M. Bhat[1], Xinxu Yuan[1], Chad Cain[2], Fadi N. Salloum[2], Pin-Lan Li[1]

[1]Department of Pharmacology and Toxicology, Virginia Commonwealth University, School of Medicine, Richmond, VA 23298
[2]VCU Pauley Heart Center, Division of Cardiology, Department of Internal Medicine, Virginia Commonwealth University, 1101 East Marshall Street, Richmond, VA 23298-0204

Running title: Medial calcification in *Smpd1* Transgenic Mice

Correspondence should be addressed to:

Pin-Lan Li, M.D, Ph.D
Department of Pharmacology & Toxicology
Medical College of Virginia Campus
Virginia Commonwealth University
Richmond, VA 23298
Tel: (804) 828-4793
Fax: (804) 828-4794
E-mail: pin-lan.li@vcuhealth.org

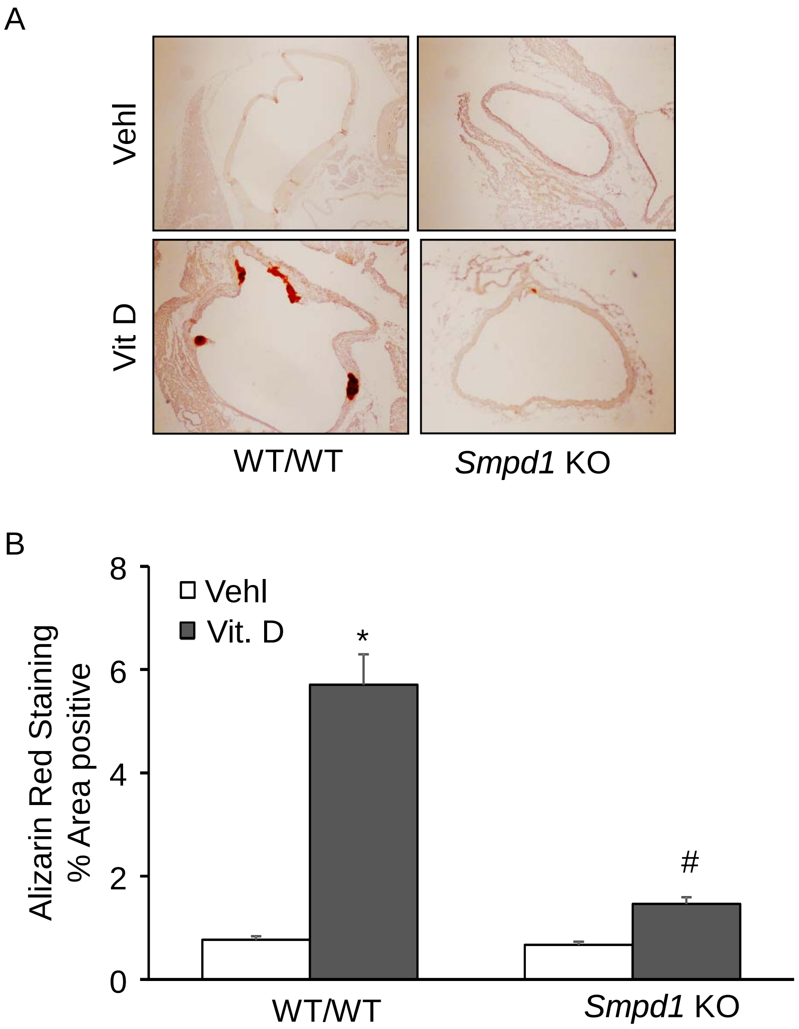

Supplementary Fig. S1 Aortic calcification in Vit D-treated *Smpd1* KO mice. Representative images of aortic sections stained by **A.** Alizarin Red S (red color) showed significantly decreased calcification in the aortic media of Vit D-treated *Smpd1* KO mice as compared to their WT/WT littermates. **B.** Summarized data showing calcification in the aortic media. Data are shown mean ± SEM of values n=3. Vehl: Vehicle; Vit D: vitamin D. * $P < 0.05$ vs. WT/WT Vehl; # $P < 0.05$ vs. WT/WT Vit D group by two-way ANOVA followed by Duncan's test.
